# Simulation and Optimization of CNTs Cold Cathode Emission Grid Structure

**DOI:** 10.3390/nano13010050

**Published:** 2022-12-22

**Authors:** Yang Zhang, Xinchuan Liu, Liye Zhao, Yuanxun Li, Zhenjun Li

**Affiliations:** 1 School of Instrument Science and Engineering, Southeast University, Nanjing 210096, China; 2 Key Laboratory of Micro-Inertial Instruments and Advanced Navigation Technology, Ministry of Education, Nanjing 210096, China; 3 CAS Key Laboratory of Nanophotonic Materials and Devices (Preparatory), National Center for Nanoscience and Technology, Beijing 100190, China; 4 GBA Research Innovation Institute for Nanotechnology, Guangzhou 510700, China

**Keywords:** carbon nanotube, field emission, cold cathode grid, CST simulation, electron transmission efficiency

## Abstract

Carbon nanotubes (CNTs) show significant advantages in the development of cold cathode X-ray tubes due to their excellent field emission performance; however, there are still some problems, such as short lifetime and the low emission current of large-area CNTs. In this paper, a front-grid carbon nanotube array model was established, and the electric field intensity near the tip of the CNTs’ electric field enhancement factor was analytically calculated. A simulation model of a CNT three-dimensional field emission electron gun was established by using computer simulation technology (CST). The effects of grid wire diameter, grid aperture shape, and the distribution of grid projection on the cathode surface on the cathode current, anode current, and electron transmission efficiency were analyzed. The aperture ratio was used to evaluate the grid performance, and the simulation results show that the ideal aperture ratio should be between 65% and 85%. A grid structure combining a coarse grid and a fine grid was designed, which can make the electric field intensity around the grid evenly distributed, and effectively increased the cathode emission current by 24.2% compared with the structure without the fine grid. The effect of grid aperture ratio on the electron transmission efficiency was tested. The simulation results and optimized structure can provide a reference for the grid design of cold cathode emission X-ray tubes.

## 1. Introduction

X-ray tubes have been applied in medical diagnoses, safety inspections, industrial non-destructive testing, and other fields [1,2,3]. The cold cathode emission X-ray tubes based on the field emission principle have the advantages of without-filament heating, small size, fast response, and high current density compared with the traditional thermal emission X-ray tubes [4], and have become one of the research focuses in the development of X-ray products in recent years [5,6,7].

Since the discovery of CNTs in 1991, as one-dimensional nanomaterials, CNTs have been considered as one of the most promising cold cathode materials due to their nano-scale and cutting-edge stable physical and chemical properties [8,9] and excellent mechanical properties [10]. It is easy to obtain the X-ray source with a small electron focal spot and programmable pulse imaging [11] because of the concentrated electron emission direction of CNTs [12], meaning femtosecond pulse electron emission can be achieved [13]. The emission current density of a single carbon nanotube can reach $10^{8}$ A/cm${}^{2}$ [14]; however, it is difficult to produce a uniform strong electric field on the surface with the increase in the emission area, which leads to the rapid decrease in the emission current density. Lei et al. [15] obtained a current density of 180 mA/cm${}^{2}$ on CNTs with an area of 0.5 mm${}^{2}$; however, the current density was only 2 mA/cm${}^{2}$ when the area increased to 1 mm${}^{2}$. Further research is necessary to obtain a carbon nanotube cathode with a large emission current density. Du et al. [16] studied the influence of grid transmission efficiency on cathode emission and electron transmission efficiency, and concluded that the maximum anode current can be obtained when the grid transmission efficiency is about 80%. Li [17] found that as the thickness of the grid increases, the cathode current increases, and the electron transmission efficiency decreases by studying the influence of the grid thickness on the field emission performance. Xu et al. [18] simulated the emission efficiency of triangular, quadrilateral, and hexagonal grids with a change of mesh size, and concluded that the best mesh shape is hexagonal, with an electron transmission efficiency of 75%. The influence of different mesh sizes of hexagonal grids on field emission performance was also analyzed [19]. Chang et al. [20] studied the influence of grid aperture structure with different sizes and shapes on field emission performance and concluded that the hexagonal grid has the highest electron transmission efficiency, while the triangular grid has the largest emission current. At present, the research on the influence of grid shape on electron transmission efficiency and emission performance is obtained by controlling the changes in emission performance caused by changes to the grid aperture’s side length, which has the limitation that the side length of different aperture shapes is the same but the aperture area size is inconsistent.

In order to improve the emission current of large-area CNTs and solve the problems resulting from the difficulties in generating a uniform electric field on the surface of CNTs, in this paper, based on the establishment of the front-grid carbon nanotube array model, we calculated the electric field intensity and field enhancement factor near the tip of the CNTs. The CST 2020 Particle Studio simulation tool was used to simulate changes to the diameter of the grid wire and the influence of the shape of the grid aperture on the cathode current, anode current, and electron transmission efficiency under the same area of the grid aperture. Finally, taking the quadrilateral grid as an example, the grid aperture ratio was proposed as the evaluating indicator, and the grid structure was optimized. On the basis of simulation, quadrilateral grids with different aperture ratios were experimentally verified.

## 2. Construction of Field Emission Model for Front Grid

### 2.1. Establishment of Structural Model

The grid is added between the anode and the cathode to reduce the field emission working voltage, forming a three-dimensional-structure field emission model. The controllable emission of the electron beam can be realized by adjusting the grid voltage [21]; however, it has different effects on the cathode electric field intensity distribution, emission current, electron transmission efficiency, and anode current.

**Figure 1:**
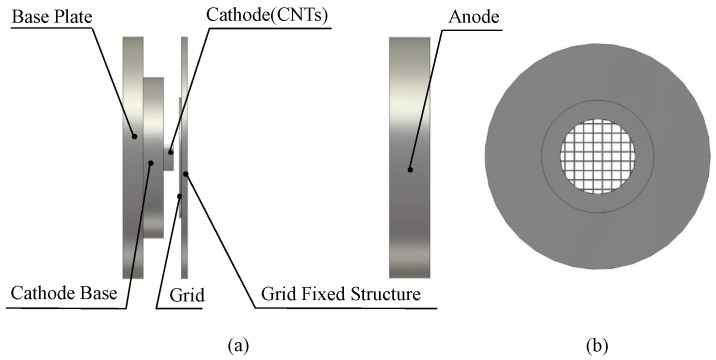
(**a**) Three-dimensional-structure model of cold cathode emission. (**b**) Grid mesh structure.

The three-dimensional emission model of cold cathode emission is shown in Figure 1a, which mainly includes a base plate, cathode base, cathode, grid, and anode. The grid is composed of a grid-fixed structure and grid, and the grid structure is precisely shown in Figure 1b.

### 2.2. Establishment of Theoretical Model

**Figure 2:**
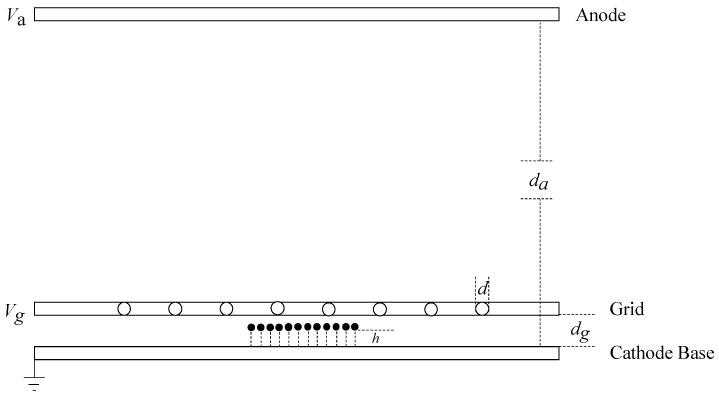
Simplified model of front-grid carbon nanotube cathode.

The electron emission ability of CNTs is determined by the electric field intensity on the tip surface. Firstly, the electric field intensity near the carbon nanotube tip without considering the contact resistance was calculated. Since most of the charge of the cathode is concentrated near the tip surface of the cathode during electron emission, a metal-suspended sphere model with −Q charge on the cathode plane can be used for simplification [22]. The front-grid carbon nanotube cathode model can be simplified as shown in Figure 2. Between the parallel infinite anode plate and cathode plate, there are carbon nanotube arrays and several infinite-charged cylinders with transverse and longitudinal diameters of *d*. The tip of the carbon nanotube is equivalent to the radius $r_{0}$ hemisphere, *h* is the height of the CNTs, and the height from the top to the cathode substrate is $h+r_{0}$. The charged cylinder is directly above the carbon nanotube, and the distance from the cathode is $d_{g}$. The distance between the anode and cathode is $d_{a}$. The cathode voltage is 0, the grid voltage is $V_{g}$, and the anode voltage is $V_{a}$. The Coulomb effect of space charge is not considered in the field emission process, and the geometric parameters of the device satisfy the following relationship: $d_{a}$ is much greater than *h* and $d_{g}$, and *h* is much greater than $r_{0}$.

According to the principle of electric field superposition, the potential near the tip surface of the CNTs is equal to the sum of the potential on the surface of CNTs when there is only a parallel plate composed of an cathode and anode, in addition to the potential generated on the surface of the CNTs by several infinitely long charged cylinders. According to the report of Lei et al. [23], the electric potential near the surface of a single carbon tube in the (2N+1)∗(2N+1) carbon nanotube array is:

(1) $$\Phi_{1}\left(r,\theta ,Q\right)=-\frac{Q(1-r/(2h))}{4\pi \varepsilon_{0}r}+E_{0}h+E_{0}r\cos\theta -\frac{p\cos\theta }{4\pi \varepsilon_{0}r^{2}}-\frac{QK}{4\pi \varepsilon_{0}L}$$

A spherical coordinate system is used, and the origin of coordinate is chosen at the center of the floating sphere. *Q* is the surface charge of the suspended sphere, $\varepsilon_{0}$ is the vacuum dielectric constant, the electric field generated by the anode is $E_{0}=V_{a}/d_{a}$, and *p* is the electric dipole moment. *L* is the distance between two CNTs, and *K* is expressed as follows:

(2) $$K=\sum_{m=0}^{N}\sum_{N=1}^{N}\left[\frac{4}{\sqrt{m^{2}+n^{2}}}-\frac{4}{\sqrt{m^{2}+n^{2}+{\left(2h/L\right)}^{2}}}\right]$$

*m* and *n* represent integers in the horizontal and vertical directions, respectively. The electric potential at the distance $r^{\prime }$ of a uniformly distributed infinitely long charged cylinder with a volume charge density of ρ and radius of *R* is:

(3) $$V=-\frac{\rho R^{2}}{4\varepsilon_{0}}+\frac{\rho R^{2}}{2\varepsilon_{0}}\ln\frac{R}{r^{{}^{\prime }}}$$

The potential at the edge of the grid mesh $\left(r^{\prime }=R\right)$ is constant to $V_{g}$:

(4) $$\rho =-\frac{4\varepsilon_{0}V_{g}}{R^{2}}$$

Substitute Equation (4) into Equation (3) to obtain:

(5) $$V=V_{g}-2V_{g}\ln\frac{R}{r^{{}^{\prime }}}$$

Near the tip of the CNT at the bottom of a single grid, the potential applied to it by the grid is the sum of the potential applied to each grid wire. The potential generated by all grid wires on the left side of a single grid is:

(6) $$\Phi_{2}=\sum_{i=0}^{M}(V_{g}-2V_{g}\ln\frac{R}{\sqrt{{\left(b+iW\right)}^{2}+{\left(d_{g}-h\right)}^{2}}})$$

*W* is the spacing between two grid wires, and *M* is the total number of grid wires on the left side of the cell. Next, arrange and obtain:

(7) $$\Phi_{2}=MV_{g}-2MV_{g}\ln R+V_{g}\sum_{i=0}^{M}\ln\left[{\left(b+iW\right)}^{2}+{\left(d_{g}-h\right)}^{2}\right]$$

Similarly, the potential generated by all grid wires on the right, front, and rear is:

(8) $$\Phi_{3}=TV_{g}-2TV_{g}\ln R+V_{g}\sum_{j=1}^{T}\ln\left[{\left(jW-b\right)}^{2}+{\left(d_{g}-h\right)}^{2}\right]$$

(9) $$\Phi_{4}=OV_{g}-2OV_{g}\ln R+V_{g}\sum_{k=0}^{O}\ln\left[{\left(a+kW\right)}^{2}+{\left(d_{g}-h\right)}^{2}\right]$$

(10) $$\Phi_{5}=PV_{g}-2PV_{g}\ln R+V_{g}\sum_{l=1}^{P}\ln\left[{\left(lW-a\right)}^{2}+{\left(d_{g}-h\right)}^{2}\right]$$

*T*, *O*, and *P* refer to the total number of grid wires on the right, front, and back of the cell, respectively, and *a* and *b* refer to the distance from the carbon nanotube to the inner back and right of the grid wire junction of the cell. According to the principle of electric field superposition, the sum of Equations (1) and (7)–(10) to obtain the electric potential near the tip of the CNT with the right number is *m*, and the backward number is *n* in the carbon nanotube array in the cell:

(11) $$\Phi_{i,j}\left(r,\theta ,Q\right)=-\frac{Q_{i,j}\left(1-r/\left(2h\right)\right)}{4\pi \varepsilon_{0}r}+E_{0}h+E_{0}r\cos\theta -\frac{p\cos\theta }{4\pi \varepsilon_{0}r^{2}}-\frac{Q_{i,j}K}{4\pi \varepsilon_{0}L}+\left(M+T+O+P\right)\left(1-2\ln R\right)V_{g}+YV_{g}$$

The expression of *Y* is:

(12) $$\begin{array}{cc} Y= & \sum_{i=0}^{M}\ln\left[{\left(mL+iW\right)}^{2}+{\left(d_{g}-h\right)}^{2}\right]+\sum_{j=1}^{T}\ln\left[{\left(jW-mL\right)}^{2}+{\left(d_{g}-h\right)}^{2}\right]+\\ \; & \sum_{k=0}^{O}\ln\left[{\left(nL+kW\right)}^{2}+{\left(d_{g}-h\right)}^{2}\right]+\sum_{l=1}^{P}\ln\left[{\left(lW-nL\right)}^{2}+{\left(d_{g}-h\right)}^{2}\right] \end{array}$$

Since the carbon nanotube is connected to the cathode, the potential of its tip surface ($r=r_{0}$) is 0. According to this boundary condition:

(13) $$Q_{i,j}=4\pi \varepsilon_{0}r_{0}\left[E_{0}h+\left(M+T+O+P\right)\left(1-2\ln R\right)V_{g}+V_{g}Y\right]{\left(1-r_{0}/2h+Kr_{0}/L\right)}^{-1}$$

(14) $$p=4\pi \varepsilon_{0}r_{0}^{3}\frac{V_{a}}{d_{a}}$$

According to the potential gradient formula E=−∇Φ, the electric field intensity near the tip surface of the carbon nanotube is:

(15) $$E=E_{r}=-{\left.\frac{\partial \Phi }{\partial r}\right|}_{r=r_{0}}^{\theta =0}=-\left[\left(3+\frac{\rho }{X}\right)\cdot E_{0}+Z\cdot V_{g}\right]$$

$\rho =h/r_{0}$ is the length–radius ratio of the CNTs. The values of coefficients *X* and *Z* are determined by the geometric parameters of the grid. The expression is as follows:

(16) $$X=1-r_{0}/\left(2h\right)+Kr_{0}/L$$

(17) $$Z=\frac{(M+T+O+P)(1-2\ln R)+Y}{Xr_{0}}$$

The electric field enhancement factor is defined as the ratio of the local electric field intensity on the cathode surface to the applied electric field intensity, and the applied electric field intensity is $E_{0}=V_{a}/d_{a}$. Therefore, the electric field enhancement factor of cathode surface is:

(18) $$\beta =|\frac{E}{E_{0}}|=3+\frac{\rho }{X}+Z\cdot V_{g}/E_{0}$$

When there is a contact resistance between the carbon nanotube and the cathode, the surface potential of the tip of the carbon nanotube is not 0 due to the voltage drop on the contact resistance. The contact barrier between the carbon nanotube and the cathode approximately satisfies the Ohm’s law [24], so the surface potential at the tip of the carbon nanotube is $V_{c}=IR_{c}$. *I* is the field emission current, $R_{c}$ is the contact resistance between the carbon nanotube and the cathode. In this case, the boundary condition is $\Phi_{i,j}\left(r_{0},\theta ,Q\right)=IR_{c}$. Similarly, the electric field intensity on the tip surface of the CNTs with contact resistance can be calculated from the above steps. The electric field intensity on the tip surface of the CNTs with contact resistance is:

(19) $$E_{a}=-\left[(3+\frac{\rho }{X}\right)\cdot E_{0}+Z\cdot V_{g}-\frac{IR_{c}}{r_{0}X}]$$

## 3. Simulation of Front-Grid Field Emission Model

### 3.1. Parameter Settings for Simulation

There is a difference between the smooth cathode surface of the simulation model in CST Particle Studio and the emission performance of the actual CNTs. In order to effectively solve this problem, it is necessary to establish a field emission model in CST Particle Studio. The surface current density of the cathode of the CST Particle Studio field emission model is given by the Fowler–Nordheim [25] expression:

(20) $$J=AE^{2}e^{-\frac{B}{E}}$$

where *J* is the current emission density, *E* is the electric field intensity, *A* is the cold cathode emission linear factor, *B* is the cold cathode emission index factor, *A* and *B* are related to the surface shape and work function of the material, respectively, and the experimental data are fitted by the data processing software to obtain $A=0.8\times 10^{-11}\;$A/V${}^{2}$, $B=6.9\times 10^{6}$ V/m. The cathode surface current emission in the simulation might have some errors with the experimental results; therefore, the relevant parameters could be corrected to ensure the accuracy of simulation results.

In the simulation model, the cathode is an elliptical cylindrical entity with a major axis of 1.3 mm, a minor axis of 0.3 mm, and a height of 0.5 mm. The distance between the grid mesh and the cathode emitter $d_{g}$ = 0.3 mm, the thickness of grid-fixed structure is 0.4 mm, the distance between cathode and anode is $d_{a}$ = 10 mm, and the experimental structure is consistent with the simulation parameter settings. The cathode was set as the emission source with a voltage of 0, and a voltage was applied to the grid to enhance the electric field intensity between the cathode and the grid to make the electrons on the cathode surface escape. The grid voltage $V_{g}$ was preset at 2000 V. If the anode voltage is too high, electrons on the cathode surface escape and the grid loses its modulation effect on cathode electron emission. Therefore, the anode voltage V${}_{a}$ was set to 7000 V.

### 3.2. Simulation Method

CST Particle Studio is an electromagnetic component for the design and analysis of accelerating and guiding charged particle beams. Before restarting the simulation engine after modeling, the full-automatic meshing program based on the expert system is used for electromagnetic calculation. The simulator supports the perfect boundary approximation feature, which significantly improves the accuracy of the electromagnetic simulation compared with the traditional simulator. The simulation steps were as follows:

(1) A three-dimensional field emission model was established, as shown in Figure 1, and the material properties were defined. The cathode, grid, and anode were defined as perfect conductor materials, and the background material was defined as a vacuum in the simulation;
(2) We set the electrode voltage. The cathode voltage was set as 0, the grid voltage was set as 2000 V, and the anode voltage was set as 7000 V;
(3) We set the cathode emission source. The particle source was a surface source and located on the upper surface of the cathode, and the *A* and *B* parameters were set according to the experiment;
(4) We divided the model grid. The overall structure was divided by automatic meshing, and the cathode grid was manually encrypted. The absolute step size of the grid in the *x*, *y*, and *z* directions was set to 0.01 mm;
(5) We set boundary conditions. It was necessary to set a certain range in each coordinate axis to surround the whole model;
(6) We set up the solver. After setting up the model, we enabled the iteration option to activate the Iterative Gun Solver algorithm, set up the particle tracking solver, and begin simulating the model.

During the simulation, the structure parameters of cathode and anode were kept unchanged, and the influence of the grid on the cathode current, anode current, and electron transmission efficiency were studied by scanning the grid structure parameters or changing the grid structure.

### 3.3. Simulation Result

#### 3.3.1. Grid Wire Diameter

**Figure 3:**
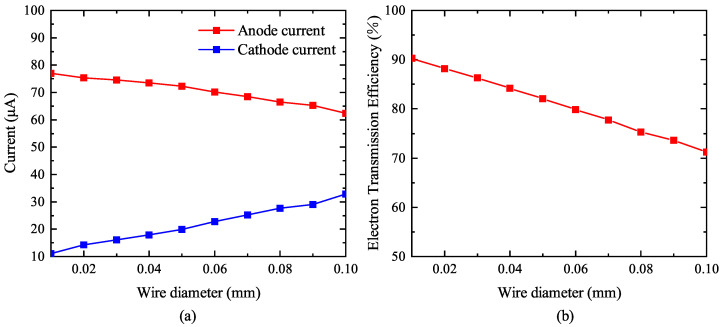
(**a**) The effect of grid wire diameter on cathode current and anode current. (**b**) The effect of grid wire diameter on electron transmission efficiency.

Taking the quadrilateral grid as an example, the grid wire spacing *W* parameter was unchanged, and the grid wire diameter *d* (d=2R) was adjusted to change the cathode surface electric field intensity strength and grid transmission efficiency. The relationship of wire diameter with cathode current, anode current, and electron transmission efficiency is shown in Figure 3. By increasing the grid wire diameter, the grid aperture becomes smaller per unit area, the area of the grid wire projected onto the cathode surface increases, and the cathode electrons are more easily captured by the grid wire after emission. It can be seen from Equations (15) and (17) that the increase in the grid wire diameter *d* leads to the increase in the electric field intensity near the cathode surface, thereby increasing the cathode emission current; however, the increase in the electrons captured by the grid wire leads to a decrease in the grid electron transmission efficiency and anode current. A smaller wire diameter can improve the transmission efficiency and anode current. In order to improve the electron transmission efficiency and anode current, the diameter of the grid wire can be appropriately reduced, but it cannot be reduced without limits. When the diameter of the grid wire is too small and the grid is hit by electrons during the emission, the grid wire is susceptible to thermal damage for a long time, and the diameter of the grid wire is also limited by the material and manufacturing technology.

#### 3.3.2. Grid Aperture Shape and Size

**Figure 4:**
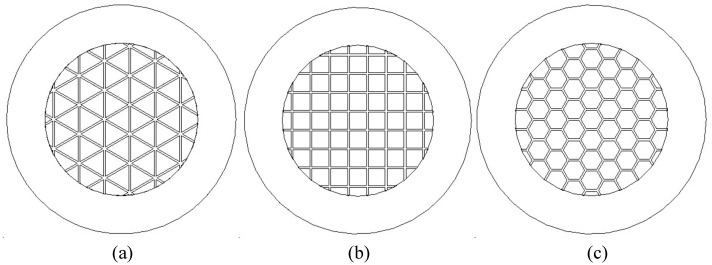
Common grid aperture shapes. (**a**) Triangle. (**b**) Quadrilateral. (**c**) Hexagon.

The common grid aperture shapes are a triangle, quadrilateral, and hexagon, as shown in Figure 4. Xu et al. [19] and Chang et al. [20] studied the influence of different grid aperture shapes on the cathode current, anode current, and electron transmission efficiency by changing the side length of the grid aperture, and came to the conclusion that the grid aperture shape with the best emission performance is a hexagon. In this paper, for each grid aperture shape, under the premise that the grid wire diameter *d* is 0.05 mm, the influence of different grid aperture shapes on cathode current, anode current, and electron transmission efficiency were studied by scanning the area of a single-grid aperture.

**Figure 5:**
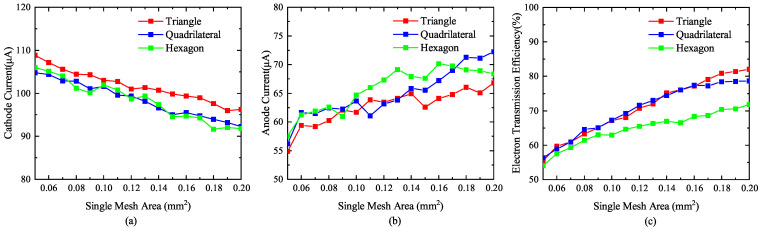
Relationship of single-grid-mesh area with cathode current, anode current, and electron transmission efficiency. (**a**) Effect of single-mesh area on cathode current. (**b**) Effect of single-mesh area on anode current. (**c**) Effect of single-mesh area on electron transmission efficiency.

Changes to the single-grid aperture area result in changes to the cathode current, anode current and electron transmission efficiency of each aperture shape, as shown in Figure 5. The cathode current of the triangular grid is larger than the quadrilateral and hexagonal grids; however, the electron transmission efficiency is lower than the quadrilateral and hexagonal grids. When the grid aperture area is less than 0.15 mm${}^{2}$, the difference between the cathode current and the anode current of the quadrilateral and hexagonal grids is not obvious. When the grid aperture area is greater than 0.15 mm${}^{2}$, the cathode current of the quadrilateral grid is greater than the hexagonal cathode current, and the anode current is greater than the hexagonal anode current when the grid area is greater than 0.18 mm${}^{2}$. The electron transmission efficiency of different aperture-shaped grids increases with the increase in the area of a single-grid aperture. When the area of the grid aperture is less than 0.16 mm${}^{2}$, the electron transmission efficiency of the quadrilateral and hexagonal grids is not significantly different. When the grid aperture area is greater than 0.16 mm${}^{2}$, the electron transmission efficiency of the quadrilateral grid is higher.

#### 3.3.3. Distribution of Grid Projection on The Cathode Surface

**Figure 6:**
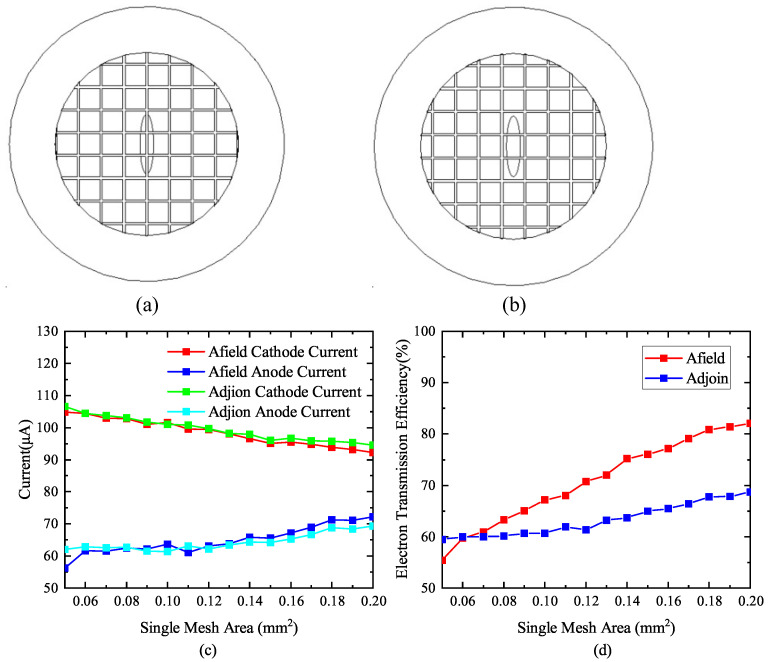
Influence of grid projection distribution on cathode surface on cathode current, anode current, and electron transmission efficiency. (**a**) Grid projection in the long axis of cathode. (**b**) Grid projection away from cathode long axis. (**c**) The influence of different grid wire projection positions on cathode current and anode current. (**d**) The influence of different grid wire projection positions on electron transmission efficiency.

Due to the limitations of manufacturing technology, the diameter of the metal grid wire cannot be processed too small. The cathode surface is an oval with a long axis of 1.3 mm and a short axis of 0.3 mm; therefore, the cathode is smaller than the entire grid. The projection position of the grid wire on the cathode surface has a great influence on the cathode current, anode current, and electron transmission efficiency. Under the condition that the remaining structural parameters remain unchanged, the projection distribution of the quadrilateral grid wire on the cathode surface was studied. Considering two special cases, the long axis of the cathode is aligned with that of the grid wire, as shown in Figure 6a, and the long axis of the cathode is located in the center of the two sections of the grid wire, as shown in Figure 6b.

The relationship of the area of the grid mesh projected at the center of the long axis of the cathode with the cathode current, anode current, and electron transmission efficiency is shown in Figure 6c,d. The cathode emission current in the two cases is not much different; however, the anode current varies with the increase in the grid aperture area. When the grid wire projection is located at the center of the cathode’s long axis, the electron transmission efficiency is not as good as that of the grid wire away from the center of the long axis. With the increase in the area of the single-grid aperture, the electron transmission efficiency of the quadrilateral grid shows an increasing trend. Even if the area of a single-grid aperture reaches 0.2 mm${}^{2}$, the electron transmission efficiency is still less than 70% when the grid wire is projected onto the long axis of the cathode. When the projection of the grid wire is located at the center of the long axis of the cathode, the electrons emitted from the cathode surface are biased towards the outer grid wire, which increases the divergent angle of the electrons. In the structure model with the focusing electrode, the electron beam is more difficult to be focused when passing through the focusing electrode.

#### 3.3.4. Grid Aperture Ratio

**Figure 7:**
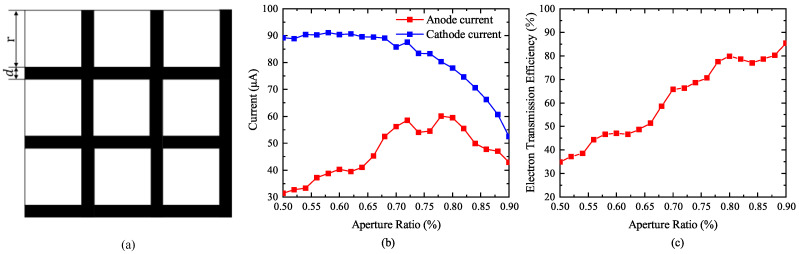
Relationship of aperture ratio with cathode current, anode current, and electron transmission efficiency. (**a**) Quadrilateral grid structure. (**b**) Effect of aperture ratio on cathode current and anode current. (**c**) Effect of aperture ratio on electron transmission efficiency.

In this paper, the aperture ratio of the grid is defined as the area occupied by the grid aperture per unit area. Taking the quadrilateral grid as an example, its structure is shown in Figure 7a. The entire grid consists of several small units, such as a single-grid aperture and two grid wires. The aperture ratio ω is defined as:

(21) $$\omega =\frac{r^{2}}{r^{2}+2rd+d^{2}}$$

*r* is the side length of a single quadrilateral grid aperture, and *d* is the diameter of the grid wire. Similarly, the corresponding aperture ratio can be obtained according to the relationship between the length of the aperture side of the triangular and hexagonal mesh and the mesh wire. When the grid aperture is quadrilateral, the grid wire diameter *d* is 0.05 mm, and the relationship between aperture ratio and cathode current, anode current, and electron transmission efficiency was studied. It can be seen from Figure 7b that the cathode current decreases with the increase in aperture ratio, and the anode current increases first and then decreases with the increase in aperture ratio. When the aperture ratio is 78%, the anode current has a maximum value. Figure 7c shows that with the increase in aperture ratio, the grid electron transmission efficiency has a maximum value of 79.84% when the aperture ratio is 80%, and the grid electron transmission efficiency keeps increasing. From Figure 7b, it is concluded that when the grid wire diameter is constant, the aperture ratio should be kept between 65% and 85%, so that a larger cathode current and anode current, as well as a higher electron transmission efficiency can be obtained.

**Figure 8:**
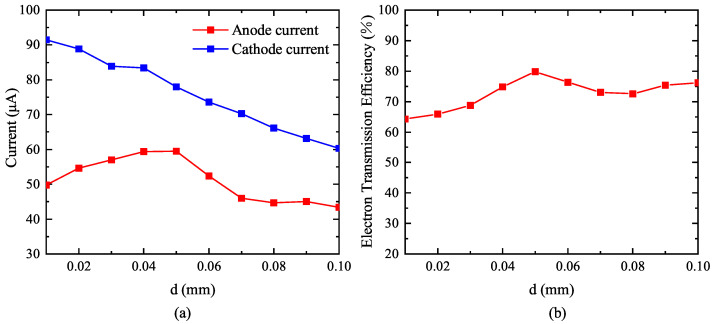
Influence of grid wire diameter *d* on current and electron transmission efficiency when aperture ratio is 80%. (**a**) Effect of grid wire diameter *d* on current at aperture ratio of 80%. (**b**) Effect of grid wire diameter *d* on electron transmission efficiency at aperture ratio of 80%.

When the aperture ratio is fixed, the side length of the grid aperture increases with the increase in the grid wire diameter. When the aperture ratio is 80%, the relationship between the grid wire diameter *d* and the cathode current, anode current, and electron transmission efficiency was studied. It can be seen from Figure 8a that the cathode current decreases with the increase in grid wire diameter, and the anode current first increases and then decreases with the increase in grid wire diameter. It can be seen from Figure 8b that with the increase in grid wire diameter, the grid electron transmission efficiency can be maintained between 60 and 80%, and when the grid wire diameter is above 0.04 mm, the electron transmission efficiency can be maintained above 70%.

#### 3.3.5. Grid Structure Optimization

**Figure 9:**
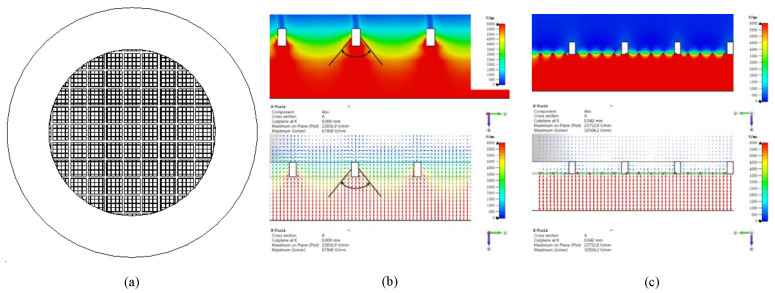
(**a**) Optimized structure of coarse- and fine-grid combination. (**b**) Electric field distribution and equipotential lines around the coarse-grid structure. (**c**) Electric field distribution and equipotential lines around the optimized structure.

The grid wire diameter is constant, and the grid mesh area increases the electron transmission efficiency so that the anode current increases. However, the electric field intensity distribution on the cathode surface is uneven due to the grid mesh area being too large; therefore, the cathode emission current and anode current are reduced. In order to increase the cathode current and anode current, a coarse grid, which has a aperture ratio of 76%, and a fine grid with diameters of 0.05 mm and 0.01 mm, respectively, are selected, and the fine grid is fixed between the coarse grid and the cathode. The structure is shown in Figure 9a. The coarse grid ensures high electron transmission efficiency and provides mechanical support for the fine grid to prevent large-area deformation damage due to electron bombardment. The fine grid makes the electric field intensity distribution between the cathode and the grid more uniform to increase the field emission cathode current. The distribution of the electric field intensity and the equipotential lines around the coarse grid is shown in Figure 9b, and the distribution of the electric field intensity and the equipotential lines around the grid structure combined with the coarse and fine grids is shown in Figure 9c. The comparison of the two figures shows that the electric field distribution on the cathode surface of the structure combined with coarse and fine grids is more uniform than that of using only coarse grids.

The field emission three-dimensional structure was used to enhance the contrast effect. The cathode is a cylinder with a diameter of 2 mm, and its height is kept unchanged at 0.5 mm. Through simulation comparison, the cathode current of the model using only the coarse grid is 404.9 μA, the anode current is 287.9 μA, and the electron transmission efficiency is 70%. The cathode current of the optimized structure model is 502.6 μA, the anode current is 295.1 μA, and the grid electron transmission efficiency is 60%. The grid structure combined with coarse and fine grids makes the electric field intensity distribution on the cathode surface and the cathode edge uniform, thus increasing the cathode emission current. Although the electron transmission efficiency decreases, the anode current increases.

## 4. Experiment Section

**Figure 10:**
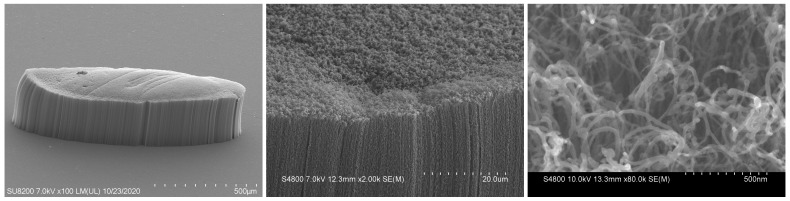
SEM images of CNTs films used in the experiment.

The effect of grid aperture ratio on the electron transmission efficiency was tested as shown in Figure 7c. The three-dimensional structure of the grid experiment is shown in Figure 1a. CNTs were fabricated by plasma-enhanced chemical vapor deposition (PECVD), and the main processes were the lithography, coating and catalytic growth of CNTs. The grown CNTs were transferred to the substrate of stainless steel through silver glue and placed in an oven at $500{\;}^{\circ }C$ for 1.5h to remove organic impurities in CNTs. SEM images of CNTs films used in the experiment are shown in Figure 10.

**Figure 11:**
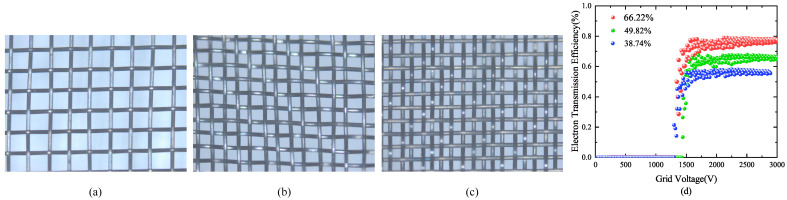
Physical diagram of grid and experimental data diagram of electron transmission efficiency. (**a**) Molybdenum mesh with aperture ratio of 66.22%. (**b**) Molybdenum mesh with aperture ratio of 49.82%. (**c**) Molybdenum mesh with aperture ratio of 38.74%. (**d**) Influence of grid voltage on electron transmission efficiency.

In the experiment, the control variable method was used to study the influence of grid aperture ratio on the electron transmission efficiency by keeping the grid width approximately consistent and the grid spacing different under the same grid material. Due to the limitations of manufacturing error, the width of the grid wire can not be guaranteed to be the same. The manufacturing of the actual wire widths were 50 μm, 47.6 μm, and 54 μm, wire distances were 208 μm, 120 μm, and 89 μm molybdenum mesh for comparative experiments, and the aperture ratios were 66.22%, 49.82%, and 38.74%. The grids are shown in Figure 11. In the experiment, the cathode voltage was 0 and the anode voltage was 7000 V. The electron transmission efficiency can be calculated by the ratio of anode current to cathode current. When the grid voltage is 3000 V, the corresponding aperture ratio grid current is 394 μA, 490 μA, 681 μA, and the corresponding anode current is 1289 μA, 925 μA, and 854 μA. The electron transmission efficiency measured in the experiment varies with the grid voltage, as shown in Figure 11d. The average electron transmission efficiency of the molybdenum mesh with a aperture ratio of 66.22% is 73.04%, the average electronic transmission efficiency of the molybdenum mesh with a aperture ratio of 49.8% is 63.48%, and the average electronic transmission efficiency of the molybdenum mesh with a aperture ratio of 38.74% is 55.12%. It can be seen from the figure that the electron transmission efficiency decreases with the decrease in the aperture ratio of the grid. To obtain a higher electron transmission efficiency, a grid with a larger aperture ratio should be selected.

## 5. Results and Discussion

From the simulation results in Figure 3, we observed that anode current and electron transmission efficiency increases as the wire diameter decreases. In order to obtain a larger anode current, the diameter of the grid wire diameter needs to be minimized.

Comparing the emission performances of different mesh grids with the same mesh area, the electron transmission efficiency of the quadrilateral and hexagonal mesh grids is not significantly different when the mesh area is less than 0.16 mm${}^{2}$, and anode current and electron transmission efficiency are the highest when the mesh area is higher than 0.16 mm${}^{2}$. It is concluded that the triangular mesh has the best emission performance and the worst electron transmission efficiency, while the best comprehensive performance is obtained in the quadrilateral grid mesh. The limitations of reference [20] is that the side lengths of different aperture shapes were the same; however, the aperture area sizes were inconsistent, meaning they were eliminated by the simulation results for the aperture shape.

The aperture ratio is used to evaluate the performance of the grid and optimize it, and a grid with an electron transmission efficiency higher than 80% is obtained when the aperture ratio is 80% and the grid wire diameter is 0.05 μm. The results of electron transmission efficiency that we obtained were better than 56% in reference [26] and 53% in reference [27]. When the aperture ratio is 80%, the electron transmission efficiency can be maintained between 60% and 80%, even if the grid wire diameter is between 0.01 mm and 0.1 mm. When designing the grid, researchers can select a higher aperture ratio, select the appropriate grid wire diameter according to the processing technology, and calculate the distance between two grid wires for grid processing.

Finally, in order to obtain a smaller grid current and larger anode current, a larger aperture ratio was chosen when designing the combined grid. The structure of the combined coarse and fine grid was designed and simulated to avoid damage due to the heat generated by the electron bombardment of the fine grid [28]. The structure of the combined coarse and fine grid was designed and simulated; however, the combined grid was not processed for the experiment due to limitations in processing technology and time.

## 6. Conclusions

In this paper, a front-grid carbon nanotube array model was established, and electric field intensity near the tip of the CNTs electric field enhancement factor was analytically calculated. Based on the model, the influence of grid wire diameter, aperture shape, distribution of the projection of grid wire on the emission surface and aperture ratio of grid on cathode current, anode current, and electron transmission efficiency was analyzed by simulation. The larger the projection of the grid wire on the cathode surface, the better the emission performance, but the lower the electron transmission efficiency. The aperture ratio is maintained between 65% and 85%, which can obtain a larger cathode current and a higher electron transmission efficiency. A structure combining a coarse grid and fine grid was proposed to improve the cathode current and anode current. Finally, the experiment of the aperture ratio of the grid on the electron transmission efficiency was carried out. It is concluded that in order to obtain a higher electron transmission efficiency, a grid with a larger aperture ratio should be selected. The above simulation and experiment can provide reference for field emission grid design.

## Data Availability

The data presented in this study are available on request from the corresponding author.

## References

[1] Críales-Vera S., Saucedo-Orozco H., Iturralde-Torres P., Martínez-Mota G., Dávila-Medina E., Guarner-Lans V., Manzano-Pech L., Pérez-Torres I., Soto M.E. (2022). Tomography and Prognostic Indices in the State of the Art of Evaluation in Hospitalized Patients with COVID-19 Pneumonia. Pathogens.

[2] Wei Z., Chu S., Huang Z., Qiu S., Zhao Q. (2020). Optimization Design of X-ray Conveyer Belt Length for Subway Security Check Systems in Beijing, China. Sustainability.

[3] Błachnio J., Chalimoniuk M., Kułaszka A., Borowczyk H., Zasada D. (2021). Exemplification of Detecting Gas Turbine Blade Structure Defects Using the X-ray Computed Tomography Method. Aerospace.

[4] Wang L., Zhao Y., Zheng K., She J., Deng S., Xu N., Chen J. (2019). Fabrication of large-area ZnO nanowire field emitter arrays by thermal oxidation for high-current application. Appl. Surf. Sci..

[5] Hong J.H., Kang J.S., Park K.C. (2017). High electron transmission coefficient on carbon nanotube emitters for X-ray sources. J. Nanosci. Nanotechnol..

[6] Kang J.S., Hong J.H., Park K.C. (2018). High-performance carbon-nanotube-based cold cathode electron beam with low-thermal-expansion gate electrode. J. Vac. Sci. Technol. B Nanotechnol. Microelectron. Mater. Process. Meas. Phenom..

[7] Park S., Kang J.T., Jeong J.W., Kim J.W., Yun K.N., Jeon H., Go E., Lee J.W., Ahn Y., Yeon J.H. (2018). A fully closed nano-focus X-ray source with carbon nanotube field emitters. IEEE Electron. Device Lett..

[8] Wickramaarachchi K., Minakshi M., Aravindh S.A., Dabare R., Gao X., Jiang Z.T., Wong K.W. (2022). Repurposing N-Doped Grape Marc for the Fabrication of Supercapacitors with Theoretical and Machine Learning Models. Nanomaterials.

[9] Minakshi M., Sharma P., Singh D., Ahuja R., Quadsia S. (2022). Activation-induced surface modulation of biowaste-derived hierarchical porous carbon for supercapacitors. ChemPlusChem.

[10] Iijima S. (1991). Helical microtubules of graphitic carbon. Nature.

[11] Li Z., Li C., Feng J., Dai Q. (2021). A Review on the Research of Cold Cathode X-Ray Tubes Based on Carbon Nanotubes. Vac. Electron..

[12] Li C., Zhou X., Zhai F., Li Z., Yao F., Qiao R., Chen K., Cole M.T., Yu D., Sun Z. (2017). Carbon nanotubes as an ultrafast emitter with a narrow energy spread at optical frequency. Adv. Mater..

[13] Djuzhev N.A., Demin G.D., Gryazneva T.A., Kireev V.Y., Novikov D.V. Investigation of the concept of a miniature X-ray source based on nanoscale vacuum field-emission triode controlled by cut-off grid voltage. Proceedings of the 2018 IEEE Conference of Russian Young Researchers in Electrical and Electronic Engineering (EIConRus).

[14] Lim S.C., Lee D.S., Choi H.K., Lee I.H., Lee Y.H. (2009). Field emission of carbon-nanotube point electron source. Diam. Relat. Mater..

[15] Lei W., Zhang X., Chen J., Zhao Z., Cui Y., Wang B. (2011). Very high field emission from a carbon nanotube array with isolated subregions and balanced resistances. IEEE Trans. Electron Devices.

[16] Du X., Zhang X., Di Y., Yu C. (2017). Simulation and Experiment of Grid Mesh Electron Transmission Efficiency Based on Carbon Nanotubes Cold Cathode Electron Gun. Chin. J. Electron. Devices.

[17] Li Q. (2018). Optimization Design of Static Digital Breast Imaging System Based on Carbon Nanotube Ray Source. Master’s Thesis.

[18] Xu W., Yuan X., Yuan J., Yang L. (2014). Mesh grid structure on field emission carbon nanotube cold cathodes. High Power Laser Part. Beams.

[19] Xu W., Yuan J., Yuan X. (2014). Hexagon Mesh Grid Structure in the Field Electron Emission of Carbon Nanotube Cold Cathodes. J. Microwaves.

[20] Chang S., Zhu Z., Lei W., Yang H. Design and Simulation of grid Structure in Field Emission X-ray Tube. Proceedings of the 20th Annual Conference of Vacuum Electronics Society (Part II).

[21] Wang X., Zha L., Qi K., Cao G., Lu R. (2019). Simulation experiment of micro-focus field emission electron gun based on OPERA. Exp. Technol. Manag..

[22] Wang X., Wang M., Li Z., Xu Y., He P. (2005). Modeling and calculation of field emission enhancement factor for carbon nanotubes array. Ultramicroscopy.

[23] Lei D., Menggen Q., Zhang H., Zhi Y. (2013). Field emission properties from a carbon nanotube array with parallel grid. Acta Phys. Sin..

[24] Lu W., Zhang S. (2012). Effect of Contact Resistance on Field Emission From Carbon Nanotube. Acta Phys. Sin..

[25] Forbes R.G. (1999). Refining the application of Fowler–Nordheim theory. Ultramicroscopy.

[26] Di Y., Wang Q., Zhang X., Lei W., Du X., Yu C. (2016). A vacuum sealed high emission current and transmission efficiency carbon nanotube triode. Aip Adv..

[27] Guo Y., Wang J., Li B., Zhang Y., Deng S., Chen J. (2022). Achieving High Current Stability of Gated Carbon Nanotube Cold Cathode Electron Source Using IGBT Modulation for X-ray Source Application. Nanomaterials.

[28] Neculaes B., Frutschy K., Cross A., Caiafa A. (2021). Experimental, analytical, and computational investigation of mesh grid thermal physics in an electron gun with dispenser cathode. Rev. Sci. Instruments.

